# Knots: Attractive Places with High Path Tortuosity in Mouse Open Field Exploration

**DOI:** 10.1371/journal.pcbi.1000638

**Published:** 2010-01-15

**Authors:** Anna Dvorkin, Henry Szechtman, Ilan Golani

**Affiliations:** 1 Department of Zoology, Tel Aviv University, Tel Aviv, Israel; 2 Department of Psychiatry and Behavioural Neurosciences, McMaster University, Hamilton, Ontario, Canada; University of California San Diego, United States of America

## Abstract

When introduced into a novel environment, mammals establish in it a preferred place marked by the highest number of visits and highest cumulative time spent in it. Examination of exploratory behavior in reference to this “home base” highlights important features of its organization. It might therefore be fruitful to search for other types of marked places in mouse exploratory behavior and examine their influence on overall behavior.

Examination of path curvatures of mice exploring a large empty arena revealed the presence of circumscribed locales marked by the performance of tortuous paths full of twists and turns. We term these places *knots*, and the behavior performed in them—*knot-scribbling*. There is typically no more than one knot per session; it has distinct boundaries and it is maintained both within and across sessions. Knots are mostly situated in the place of introduction into the arena, here away from walls. Knots are not characterized by the features of a home base, except for a high speed during inbound and a low speed during outbound paths. The establishment of knots is enhanced by injecting the mouse with saline and placing it in an exposed portion of the arena, suggesting that stress and the arousal associated with it consolidate a long-term contingency between a particular locale and knot-scribbling.

In an environment devoid of proximal cues mice mark a locale associated with arousal by twisting and turning in it. This creates a self-generated, often centrally located landmark. The tortuosity of the path traced during the behavior implies almost concurrent multiple views of the environment. Knot-scribbling could therefore function as a way to obtain an overview of the entire environment, allowing re-calibration of the mouse's locale map and compass directions. The rich vestibular input generated by scribbling could improve the interpretation of the visual scene.

## Author Summary

Exploration is a central component of human and animal behavior that has been studied in rodents for almost a century. It is presently one of the main models for studying the interface between behavior, genetics, drugs, and the brain. Until recently the exploration of an open field by rodents has been considered to be largely stochastic. Lately, this behavior is being gradually deciphered, revealing reference places called home bases, from which the animals perform roundtrips into the environment, tracing well-trodden paths whose features contribute to our understanding of navigation, locational memory, cognition-, and emotion-related behavior. Using advanced computational tools we discover so-called *knots*, preferred places visited sporadically by mice. Mice perform in these places twists and turns. The measurement of speed on the way in and out of knots reveals that they are attractive for the mice. Knot formation is enhanced by stress, suggesting that stress-related arousal assigns these locales with a special significance that is reinstated by subsequent visits to them. The twists and turns could provide the mouse with multiple views that turn knots into navigational landmarks as well as with rich vestibular input that might improve the perception and subsequent interpretation of the visual input.

## Introduction

When introduced into a novel environment rats establish in it a preferred place characterized by the highest frequency of visits, by the highest cumulative dwell time, by an upper bound on the number of stops per roundtrip performed from it, by low outbound trajectory speed and high inbound trajectory speed, with the speed relationship reversed in later stages of the session [1]–[2]. The existence of highly organized behavior across the whole arena in reference to this so-called *home base* [3] illustrates the influence a preferred place might have on the overall organization of exploratory behavior and prompts the search for other types of preferred places that may have a similar (or different) influence on the organization of exploratory behavior.

In contrast to the situation with rats, there is an ambiguity regarding the establishment of home bases in mice: while some studies mention home bases, their establishment is mostly of a low occurrence in the forced exploration setup. Even though mice readily establish home bases near physical objects [4]–[6] and near nesting material [7], they are reported to fail to establish distinct home bases during forced exploration of a relatively featureless environment [7]–[8].

When mice used as a control group in another study were injected with saline and placed in the exposed portion of a large open field arena, they established in it preferred places, typically not more than one per session, which they visited sporadically, tracing in them a tortuous path full of twists, turns, and bends that looked like a knot. We termed these places *knots*, and the behavior performed in them - *knot-scribbling*. This knot phenomenon, which appeared in its full blown form in the saline-injected mice, was subsequently uncovered, albeit in a less striking form, also in intact mice. In the present study we describe the full-blown knot phenomenon in saline-injected mice and only then verify the existence of knots in intact mice.

To uncover knots we utilize a basic feature of the locomotor path – its curvature. Depending on the size of the window that is used for measuring path curvature this measure discloses various features of path texture, from overall large-scale curvature to fine-grained tortuosity [9]. In the present study we use a window size that makes the fine-grained tortuosity of the path as conspicuous as possible (see *Materials and Methods*, [9]). Curvature has been measured previously only as a cumulative measure of paths without coupling a particular degree of curvature to particular topographical locations [9]–[10]. To uncover knots we first calculate fine-grained path curvature for each data point on the path traced by the mouse. Then we partition the arena into small 5×5cm unit areas and summate, for each unit area, the curvature of the path included within that unit area. The magnitude of path curvature per unit area is then color coded in a visualization of the paths, thus highlighting the unit areas that are marked by the highest curvature. Having uncovered a high curvature locale we then design and use algorithms that define its boundaries and quantify some of its features. Finally, we ask whether the knots we discover are also endowed with the classical home base features, and consider their function.

## Results

### Isolating knots

**Figure 1:**
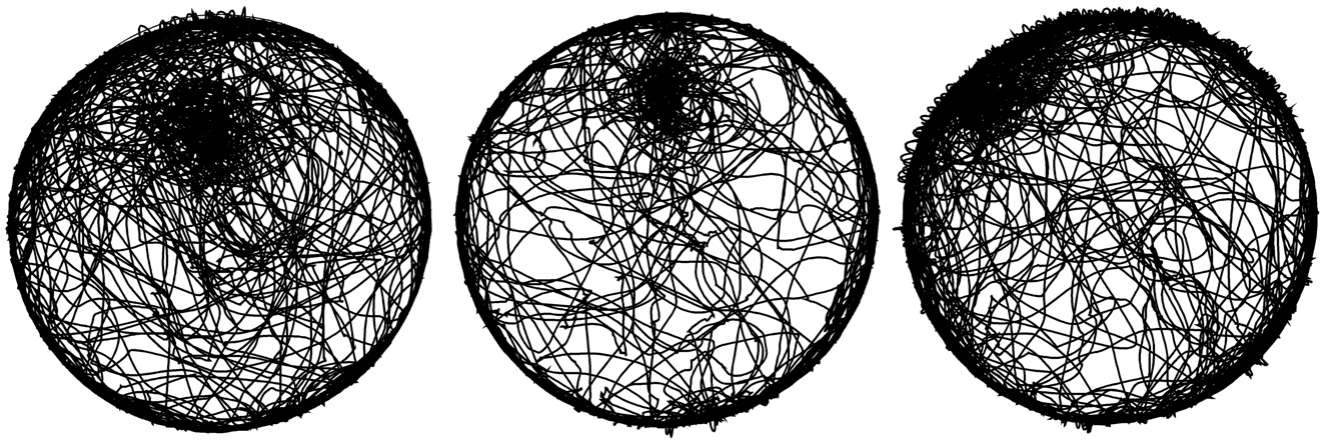
Paths of 3 representative mice during a 55min session. The mice were released at the same location, at the 12 o'clock position 50 cm away from the wall. Dark patches reflect high path density.

**Figure 2:**
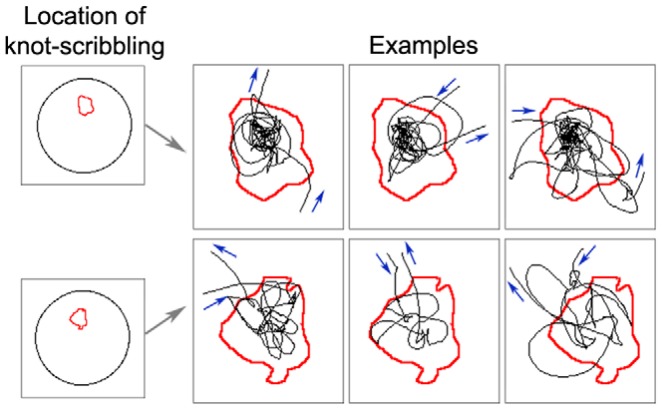
Illustrations of the knot-scribbling performed by two representative mice in their preferred places (knots). The leftmost figure in each horizontal line of figures shows boundaries of a preferred place within the arena; the rest of the figures in each horizontal line represent close-ups of 3 separate entries into that place. Red line presents the boundary of the knot, computed across the whole session, and black line presents the path. Blue arrows indicate the entry and exit paths into and out of the knot.

Observations of saline-injected mice revealed that when mice were released into the arena at a distance from the wall they showed a preference for a selected place often at the location of release, but also at other locations. The phenomenon is illustrated in the behavior of 3 mice, two of whom established a preferred place around the location of their release, and one that established such a place near the wall (Figure 1). A close-up examination of the behavior revealed that during their sporadic visits to these places the mice traced in them a tortuous winding path full of twists, turns and bends, reminding of knots. The behavior performed within the knots was termed *knot-scribbling* (Figure 2).

**Figure 3:**
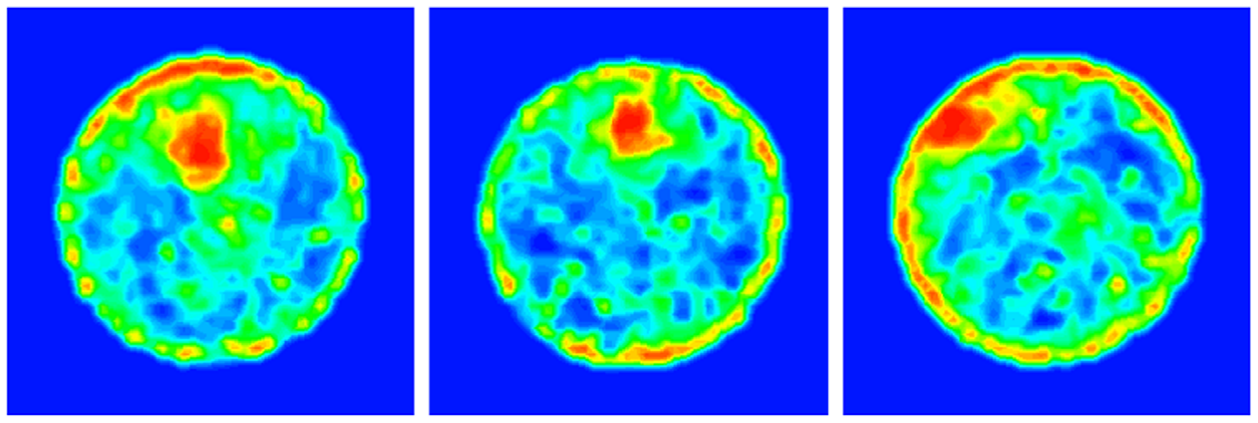
Contour plots of the sessions of 3 representative mice (see Figure 1). The colors represent path curvature measured with a 20cm window. Locations without paths and locations with low path curvatures are shown in blue, locations with medium curvature are shown in green, and locations with high path curvature are shown in red.

To capture the knots and map their boundaries we produced visual representations that highlighted places with knots by computing momentary path curvatures for all locations in the arena, and obtained a contour plot that mapped their boundaries (see *Materials and Methods*). Creating visual representations of locations with knots. Highlighting locations with high path curvatures (as in Figure 3) was accomplished in the following way: after calculating path curvature for each data point, we divided the arena into 5×5cm squares, and computed quantile 95 of all path curvatures belonging to each square (see *Materials and Methods*).

A matrix whose cells present these values of curvature per each square in the arena portrayed the level of curvature across the arena topography. To highlight areas characterized by similar curvature we used a spatial smoothing algorithm with a Gaussian kernel (see *Materials and Methods*). The matrix containing the smoothed values of curvatures was then visualized by using a 2D contour plot (see Figure 3). In this map the colors represented path curvature measured with a 20cm window. Empty locations (without paths) and locations with low path curvature were colored in blue, and locations with high path curvatures were colored in red (for plots of all mice see Figure S1).

Figure 3 shows the curvature contour plots for the sessions illustrated in Figure 1. As shown, high path curvature locations coincided with the dark patches presented in Figure 1. High curvature patches evident along and near the wall (maximal distance 30 cm), reflected u-turns performed by the mice, often during repeated attempts to jump on the wall while walking back-and-forth along a short wall section.

### Mapping knot boundaries

**Figure 4:**
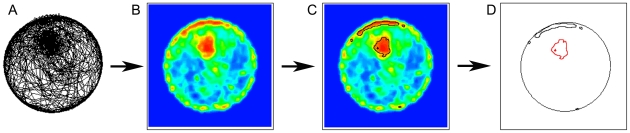
An illustration of the operation of the algorithm for mapping the boundaries of knots. A. A selected mouse-session cumulative path plot. B. A contour plot of fine-grained curvature topography of the same session. C. High curvature patches are circumscribed by a black boundary. D. The boundaries of u-turns are drawn in black. These patches of u-turns are filtered out. The boundary of the knot is singled out and drawn in red. In both B and C the colors represent path curvature measured with a 20cm window. Curvature values are represented by the same colors as in Figure 3.

To map the boundaries of knots we developed an image processing algorithm identifying areas filled with the red color that signified high curvature patches, and then filtered out the patches of u-turns along the wall (Figure 4). The algorithm provides a numerical specification of the Cartesian coordinates of the knot boundary.

### Places with high path density are not necessarily associated with knots

**Figure 5:**
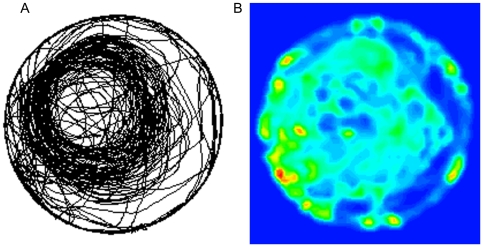
Path density and path curvature are two independent measures. A. The path plot of a mouse-session portrays a circular bundle of paths. B. The contour plot of the same circular bundle of paths does not pick up the high path density patches because path curvature is of a different scale. Curvature window size and colors are as in Figure 3.

A high density of paths does not imply the presence of a knot. Figure 5 presents an example of a path traced by a c57 mouse across a session (the example is taken from an unpublished study). As illustrated, the high density of the circular paths, which was conspicuous in the path plot, did not show at all in the visualization generated by our curvature algorithm. This is because the large-scale curvature of this path is not captured by an algorithm designed to capture finer-grained scale curvature of a tortuous path. More generally, high density patches may include frequently-visited-places, heavily trodden paths, or sharp turns traced within a circumscribed area. These 3 types of “preferred” portions in the arena can not be distinguished from each other on the basis of the density of the path plots included in them. The only way to identify knots is by singling out locations with a high density of sharp turns. This is accomplished by our algorithm, which focuses only on curvature at a particular scale much like a roentgen image that represents the transparency of the tissue to radiation at the expense of sacrificing all other detail thus bringing into high relief the bright patches of high density tissue.

### Visits to knots

**Figure 6:**
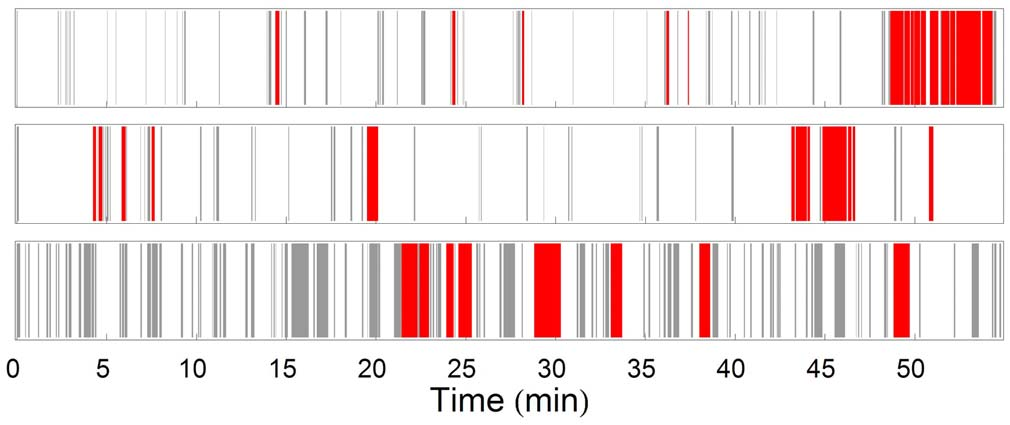
Timing and frequency of visits to knots in 3 representative mouse-sessions. Each horizontal line represents the visits of a selected mouse. Regular visits, not involving scribbling, are shown in gray and knot-scribbling visits are shown in red. Vertical bars' widths represent the durations of the visits.

Knots were established at the location of the release of the mouse into the open field in 25 out of 41 mouse-sessions, in which knots were established. Mice left the release location almost immediately after being placed in it (3.8–34.3 sec), implying that the subsequent selection of this place for knot-scribbling did not depend on a long initial stay in it. The knots were visited for short periods of time (1.1–5.4 sec): unlike home bases, these places are not characterized by long visits. As illustrated in Figure 6 for the same 3 mice presented earlier, they all left the place where the knot-scribbling was later performed a short time after the start of the session, and performed knot-scribbling in it sporadically across the whole session (for timing and frequency of knot-scribbling across sessions for all mice see Figure S2). The visits to knots took place quite frequently - at a median of 5.5 minutes intervals, with knot-scribbling displayed during only 4.9%–24.3% of the visits.

### Knot location across sessions

**Figure 7:**
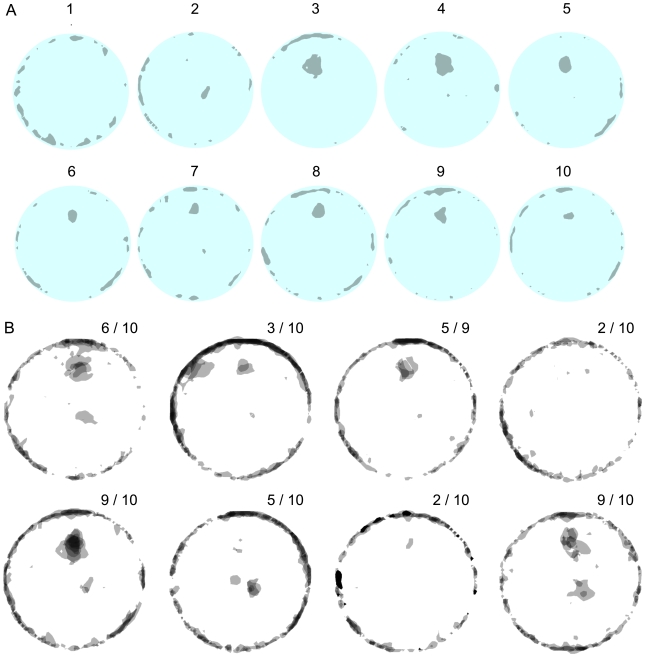
Knot locations in open field arena across sessions. A. Dark patches represent places with high path curvatures (knots and u-turns) established by a selected mouse across 10 successive sessions. The patches are shown in gray and the arena in cyan. Sessions are numbered. B. A superposition of all the patches, representing all knot and u-turn locations in the arena during 10 successive sessions, per mouse, for the 8 mice. Each circle represents 10 successive mouse sessions with knots superimposed across 10 sessions for each tested mouse. The shades of gray of the patches represent the number of superimposed patches, which in turn represent the number of knots established in the same location across the 10 session experiment: the darker the patch the higher the number of knots established in that location. The indices in the right upper corner indicate the number of sessions during which the mouse established a knot as a proportion of the total number of recorded sessions. One mouse had only 9 recorded sessions because of a technical failure.

Figure 7A presents the silhouettes of all the places that are characterized by a high curvature (including knots) in 10 successive sessions of one mouse. This mouse established a knot in the same location, at the 12 o'clock position 50 cm away from the wall, across sessions 3–10; near the center of the arena in session 2, and no knot at all in session 1. In order to examine the degree of overlap in knot locations across sessions, the 10 successive sessions of each of the mice were superimposed on each other (see Figure 7B). The frequency of overlaps is represented by grey level intensity. All knots, including those touching the wall, extended into the central portion of the arena (more than 30 cm away from the wall). Almost all mice had knots in one or two well-defined places across at least several sessions. Some mice (e.g., Figure 7B third mouse from left top row) established the knot always in the same location (at the 12 o'clock position 50 cm away from the wall), whereas others (e.g., Figure 7B fourth mouse from left bottom row) alternated across sessions between two locations (one at the 12 o'clock position 50 cm away from the wall and the other near the center of the arena). All mice established, however, only one knot per session (see also Figure S1).

### Do knots have home base properties?

#### 1. Dwell time and number of visits

**Figure 8:**
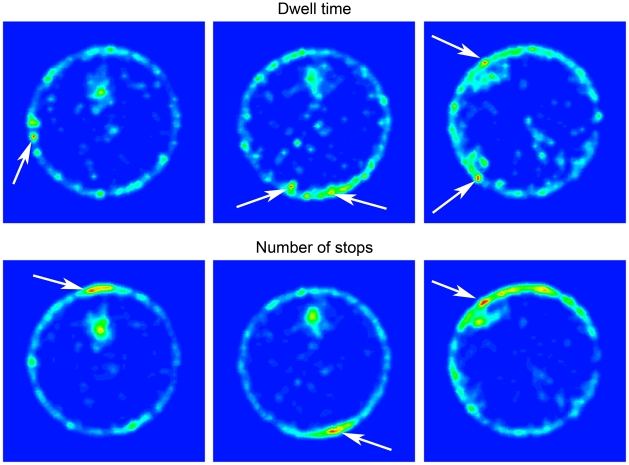
Contour plots representing cumulative dwell time (top row) and overall number of visits (bottom row) per location in the same 3 selected mice. *Top row*: Locations without paths and locations with low dwell time are colored in blue, locations with medium cumulative dwell time are colored in green, and locations with the highest dwell time are colored in red. Arrows indicate highest dwell time locations. *Bottom row*: Same as top row for number of visits per location.

To see whether home base locations coincide with knot locations we inspected the places characterized by two home base properties - the highest number of stops and the highest cumulative dwell time (see Figure 8 and Figures S3 and S4).

**Figure 9:**
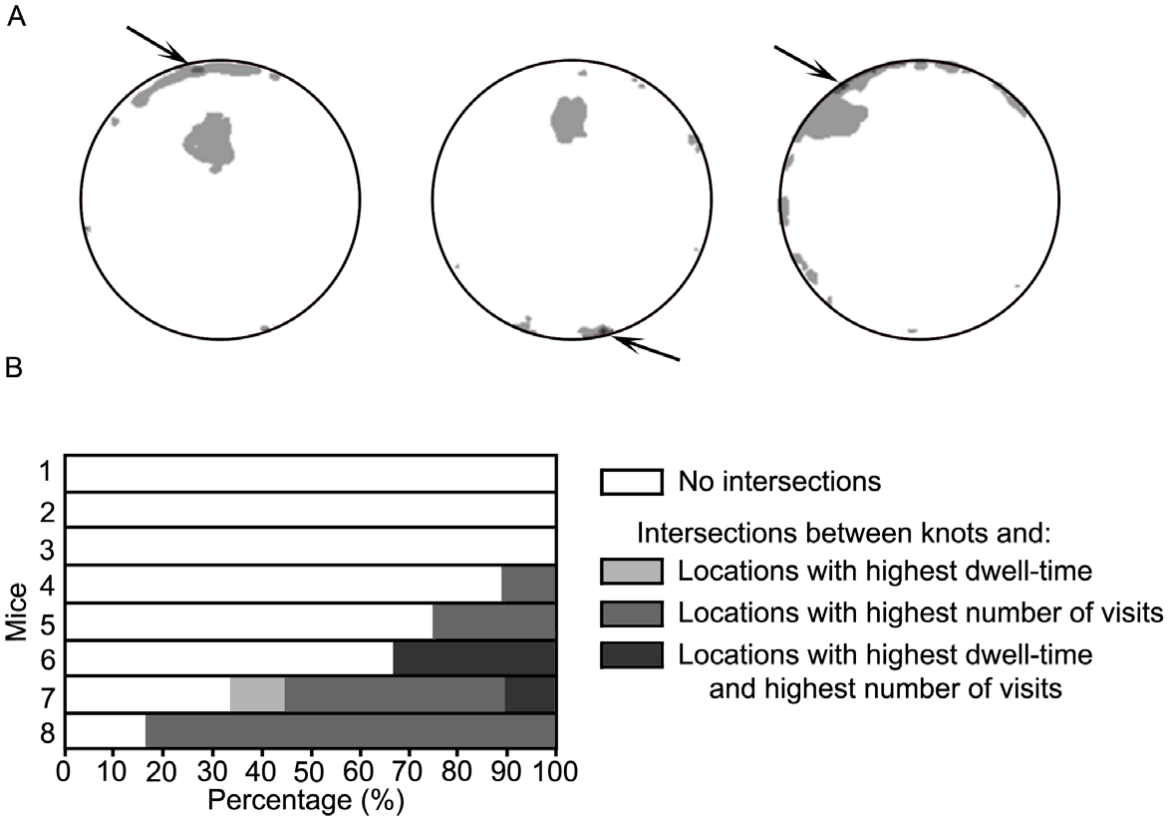
The degree of overlap between locations marked by high curvature, locations marked by the highest number of stops, and locations marked by the highest cumulative dwell time. A. The degree of overlap between these 3 types of locations in the 3 selected mice. B. The degree of overlap between the 3 types of locations in all mice. For each mouse, the occurrence of every type of overlap was calculated as the percentage of the total number of sessions in which that mouse established a knot. Each row represents the types and percentages of overlap for each of the mice.

Figure 8 further illustrates that highest dwell time and highest-number-of-visits locations were mostly situated near the wall, unlike the knots that were mostly situated away from the wall. As illustrated in Figure 9A, locations characterized by all three criteria did not appear in the knots located away from the wall. In most cases there were no intersections between the knots and either the highest dwell time places or the most visited places (see Figure 9B). Only two mice established the knot at a location characterized both by the highest dwell time and by the highest number of visits.

#### 2. Outbound/inbound speed ratio

**Figure 10:**
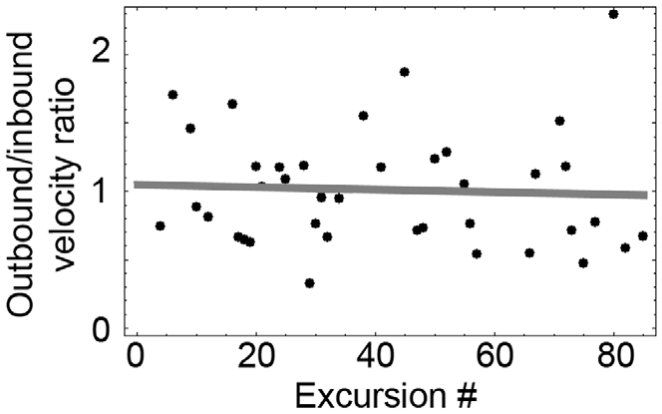
Fitting a linear regression to the outbound/inbound speed ratio in trajectories leading out of knots and into knots. Ratio values per visit are presented by black dots; fitted linear regression is presented by a gray line.

To examine whether knots were characterized, like home bases [3], by low-outbound/high-inbound speeds [1]–[2] we first examined whether the ratio between these 2 speeds changed across the session. Calculating these ratios, plotting them against the ordinal number of the excursion from the knots and fitting a linear regression to these ratios (Figure 10) revealed that the ratio remained constant across the session (Mean t-test, p-value = 0.526621).

**Figure 11:**
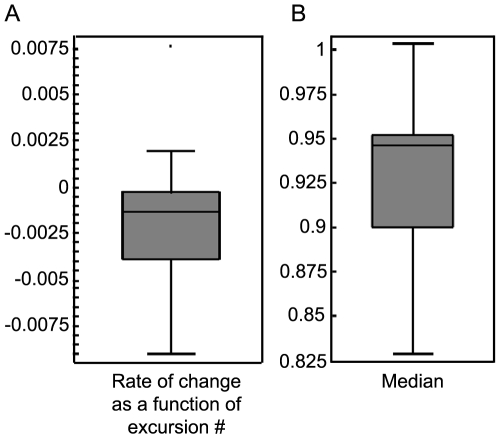
Boxplot summaries of the outbound/inbound speed ratio. A. Rate of change of the outbound/inbound speeds' ratio within session as a function of excursion number. B. Median value of the outbound/inbound speed ratios per session.

Since there was no change in the ratios (Figure 11A), it became possible to pool all the ratios across sessions and examine the median ratio per session for each mouse. The median ratio of outbound/inbound speeds per session (Figure 11B) was significantly lower than 1 (95% confidence interval for median ratio of outbound/inbound speeds per session was (0.906028, 0.986511)), implying that the outbound speeds were lower than the inbound speeds.

### Presence of knots in intact mice

**Figure 12:**
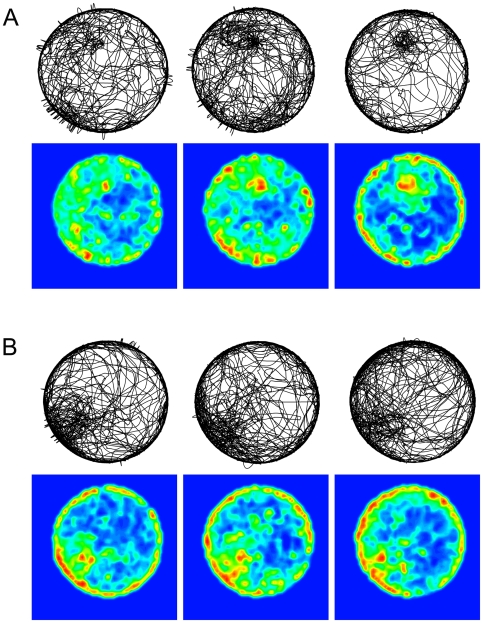
Path plots and corresponding contour plots of sessions of selected intact mice. A. Knots established between 12 o'clock positions and center in three intact mice placed in the center area. B. Knots at about 8 o'clock positions away from wall in three intact mice placed near the wall at the 12 o'clock position.

To examine whether uninjected mice also established knots, and whether the location of introduction influenced knot frequency, we performed a subsequent experiment using intact mice that were either placed near the wall or in the center. The center-placing location was the same for the intact and for the injected mice (see *Materials and Methods*). As shown (Table 1), when the mice were released in the center of the arena, only a quarter of the intact mouse-sessions, as opposed to a half of the saline-injected mouse-sessions included a knot. Placing the intact mice near the wall limited the establishments of knots even further to only an eighth of the sessions. As shown, more than half of the knots were established in the intact mice that were placed in the center at the point of introduction into the arena, a similar proportion to that observed in the injected mice (Table 1, Figure 12A). In contrast, none of the intact mice placed near the wall established a knot in that place (Table 1, Figure 12B). The intact mice knots are not as striking as their counterparts in the injected mice, but nevertheless conspicuous, consisting of the same tortuous paths highlighted by the tortuosity algorithm. As illustrated in Figure S5, knots were also established during mouse exploration of a large open field arena allowing free access to the mouse's home cage (for the detailed description of the experiment see [11]). It may thus be concluded that intact mice do establish knots. The knots are established regardless of a saline injection, and are, therefore, natural ethological building blocks of mouse exploration. Their formation is enhanced by the injection but does not depend on it.

**Table 1:** Establishment of knots in injected and uninjected mice placed at a 50cm distance from the wall (Center) or near the wall (Wall).

	Saline injections (Center)	No injections (Center)	No injections (Wall)
Number of mice	8	9	9
Number of sessions	10	7	7
Total number of analyzed sessions	79	63	63
Number of knots established at the place of the introduction into the arena	25 (60.9%)	9 (56.3%)	0 (0%)
Total number of knots	41 (51.9%)	16 (25.4%)	8 (12.7%)
Different mice showing knots	8 (100%)	7 (77.8%)	5 (55.6%)

Percentages of the established knots (row 5) are calculated as a percentage out of the total number of mouse-sessions in this study (row 3). Percentages of the knots established at the place of the introduction (row 4) are calculated as a percentage out of the total number of knots (row 5).

## Discussion

In this study we uncover a new type of preferred places called knots, established by mice during exploratory behavior of an open field arena. The behavior generating these places, knot-scribbling, is performed by the mice during sporadic recurrent visits to the knot during the session. Knots are also characterized by a high path density, by frequent visits that do not involve scribbling, and by a higher speed on the way into the knot than on the way out of it. There is at most one knot per session and it is established in a circumscribed location having distinct boundaries. Knots are often established across successive sessions in the same place, but also in two or even three different places. They can be isolated solely on the basis of the curvature of the paths included in them. To make them “float” out of the background of paths we had to design and use an algorithm that calculated path curvature at the fine-grained scale that characterizes them. The algorithm scans the whole arena, highlighting locations with high cumulative path curvature and identifying their boundaries.

Unlike home bases, knots are neither characterized by maximal cumulative dwell time, nor by maximal number of stops. The reversal of the outbound-inbound speed ratio characterizing rat home base behavior across sessions [1] is absent with regard to knots: while in rats habituation across sessions turns the home base from an attractor to a repeller, in mice knots remain attractive both within and across sessions. The constancy of the speed ratio cannot be ascribed to an absence of locational memory in C57 mice, since they were shown to keep a record of their visiting history to places [12]. Rushing to knots is thus mediated by a different mechanism than rushing to the home base.

Mice tended to perform knot-scribbling at the point of introduction into the arena although they left these places immediately after being placed there. A preference for the point of entry, expressed by a longer-than-expected dwell time spent in the entry quadrant has been described in rats [13]. Perhaps also in mice the arousal associated with the moment of introduction into the arena creates a memory and a preference for that place. This could also explain why placing the mouse near the sheltering wall is not followed by knot establishment in that place. The contingency between an injection and a forced introduction into the exposed center could further increase the mouse's arousal, explaining the high knot frequency in injected-and-center-placed mice (51.9%), the lower frequency in only-center-placed (25.4%) and the lowest frequency in near-wall-placed mice (12.7%).

Deciphering the function of knots will require extensive experimental manipulations, not available in this, mostly descriptive, study; some hypotheses may, however, be appropriate: having established a place of significance in an otherwise empty arena the mouse marks this place with twists, bends and turns that provide it with multiple, almost simultaneous, views of the explored environment, all from a single reference place. Perhaps while scribbling at the knot the mouse recalibrates its locale map and compass directions, in much the same way as a robot designed by Steinhage and Schoner [14] achieves, during sporadic visits to a central place in an explored environment these diverse functions. A recurrent resetting of the *idiothetic* information gained by the mouse's own movement, by using *allothetic* information that is independent of the mouse's movement has been hypothesized by many (e.g., [15]). Based on these navigational considerations Mittelstaedt [16] predicted at the time a “still undiscovered performance” aimed at recalibrating and resetting the navigational system. The behavior at the knot could well fulfill that predicted function.

It has recently been demonstrated experimentally that a short vestibular stimulus can influence and even improve the perception and subsequent reconstruction of a much longer lasting visual stimulus [17]. In an empty arena devoid of proximal cues, the rich vestibular and proprioceptive inputs generated by the scribbling could improve the mouse's view of the environment and more generally enhance or even embody for the mouse the memory and significance of this place by tagging it with a place-specific proprioceptive and vestibular signature.

The structure of the knot, its effect on the behavior of the mouse when away from it, and the context of its formation, all rule out the possibility that the knot replaces a home base, or is interchangeable with a home base, or fulfills a similar function to a home base. A recent study reveals that when mice are exposed to a setup including a shelter and a free access to a large open field arena they demonstrate the full blown home base phenomenon [11] yet may concurrently establish a knot within the arena (Figure S5). Knots and home bases are thus two relatively independent types of preferred places.

## Materials and Methods

### Subjects

Saline-injected mice: 8 experimentally naive 8–11 week old male C57BL/6J mice (Charles-River, Canada) were used.

Intact mice: 18 experimentally naive 8–11 week old male C57BL/6J mice (Charles-River, Canada) were used.

All mice were individually housed in a temperature controlled colony room (22°C) under a 12h light-dark cycle, with free access to food and water. Mice were allowed to acclimatize to the colony room for two weeks following arrival and were handled two minutes daily for 7 days before the start of the experiment. All treatment and testing was conducted during the light hours.

### Ethics statement

Animals were housed and tested in compliance with the guidelines described in the Guide to the Care and Use of Experimental Animals (Canadian Council on Animal Care, 1993).

### Apparatus

The large open field was a 220cm diameter circular arena with a non-porous gray floor and a 50cm high, primer gray painted, continuous wall. Several landmarks could be seen near the walls of the room where the arena was located. In particular, there was a table and a stand on which the lights were fixed. The arena was recorded using a resolution of 30 samples per second. The animal's movement was tracked using Noldus EthoVision automated tracking system [18]–[19].

### Design and procedure

Saline-injected mice: On the day of testing, animals were weighed, transported in their home cages to an adjoining non-colony testing room, and administered a sub-cutaneous saline injection. Immediately afterwards, mice were placed within a small opaque box 50 cm from the wall of the circular open field arena at the 12 o'clock position. After 20 seconds the box was lifted, and a 55-min session began. Each mouse was videotaped for 10 sessions (2 sessions a week). Animals were tested during the light phase of the diurnal cycle.

Intact mice: Same as above except that the stage of injection was skipped, and mice were placed either near the wall, at the 12 o'clock position (n = 9), or 50 cm away from the wall at the 12 o'clock position (n = 9). The experiment with the intact mice was carried out in the same setup, after the completion of the experiment with the saline-injected mice.

### Analysis of behavior

The raw data obtained from the tracking system were smoothed using a specialized algorithm implemented in the stand-alone program “SEE Path Smoother” [20]. This procedure produces reliable estimates of momentary speeds during motion (momentary speeds during arrests were defined as zero). As was previously shown, rodent locomotor behavior consists of two distinct modes of motion – progression segments and lingering episodes [21]–[23]. During progression segments, the animals traverse relatively large distances attaining relatively high speeds. During lingering episodes the animals stop and perform scanning movements, while staying in a circumscribed neighborhood. Segmentation of the smoothed path into progression segments and lingering episodes was done using the EM algorithm [24] with a two-gaussians mixture model ([21],[23] http://www.tau.ac.il/~ilan99/see/help/). The development of the new algorithms and endpoints described in the results section was done using the Mathematica software (Wolfram).

#### Computation of path curvature

**Figure 13:**
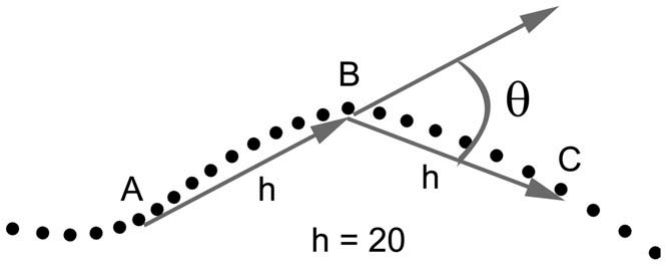
Computation of path curvature. Example of the computation of the curvature of the path at data point B. The curvature in degree/cm is defined as θ/(2*h*) where θ denotes direction change and *h* denotes distance between the point B to points A and C.

A measure of curvature was computed for all data points obtained from the session. Curvature was measured as described previously ([9]–[10]; for illustration, see Figure 13): For each data point B, find the data points that occur immediately prior to and after that point (points A and C respectively) that are at least *h* cm distant from B (*h* = 20cm). The direction change *θ* between lines AB and BC is the direction change at point B. This direction change is thus defined as zero when A, B and C are on a straight line (i.e., the animal is moving straight forward). Positive direction changes imply curving to the left while negative direction changes imply curving to the right. The curvature in degree/cm for data point B was defined as *θ*/(2*h*). Note that the measure we use is not the curvature of the graph as defined mathematically, but rather a monotone transformation of it per a fixed *h*.

#### Defining knot contours in mice

**Figure 14:**
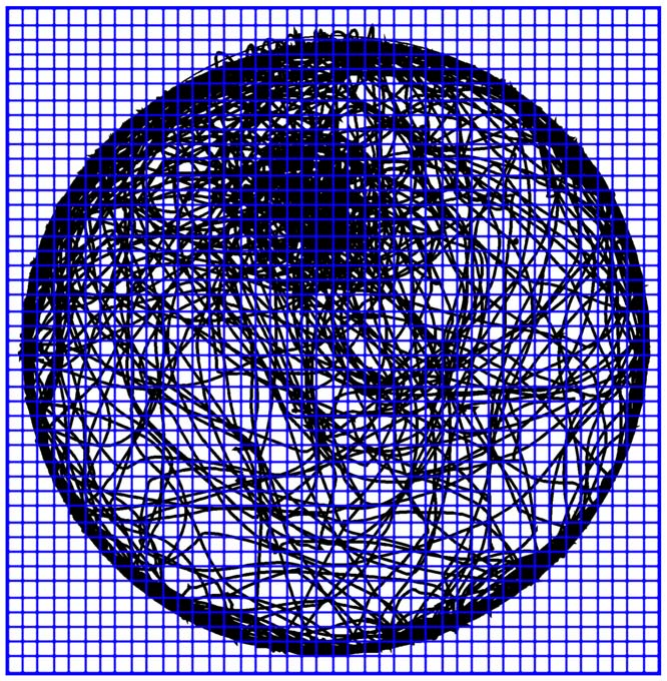
A grid of 5×5cm squares superimposed on the path traced by a mouse during a 55min session.

The algorithm for defining the knot contours was as follows:

1. Curvature was computed for all data points obtained from the session.
2. The arena was divided into 5×5cm squares (see Figure 14), for each square all data points belonging to it were collected and a quantile 95 of the absolute curvatures of these data points was calculated. The computation was performed only on the cells containing more than one visit (a visit was defined as a continuous path starting from the moment the animal entered the grid cell and ending at the moment the animal exited that cell). Grid cells containing one or zero visits received a zero value.
3. In order to highlight areas characterized by similar curvatures, we used a spatial smoothing algorithm with a Gaussian kernel (MatLab R2007a). The obtained matrix containing smoothed values of curvatures was used to create a 2D contour plot, and then the plot's contour lines were used in order to find the areas characterized by high path curvatures.

Examination of the paths traversed by the mice during the session revealed that some of the patches with high path curvature demarcated u-turns performed by the mice in close proximity to the wall. Since u-turn locations did not exceed a distance of more than 30 cm from the wall, knots were defined as patches of high curvature whose boundary extended for more than 30 cm away from the wall (see for example rightmost session in Figure 3).

### Statistical methods

#### Computing the delay until the first exit from a knot

To examine whether the establishment of a knot at the location where the mouse was placed was a result of a prolonged initial stay in it we measured the delay until the first exit from that place. Therefore, only sessions during which mice established a knot at that location of entry were used in the analysis. A median value of the duration of the delay until the first exit from a place later established as a knot was computed for each mouse across all sessions during which it indeed established a knot.

#### Examining maximal outbound/inbound speeds' ratios

For each preferred place a circle of minimal area circumscribing it was computed. Maximal inbound speed was computed as a quantile 99 of speeds exhibited along the inbound path located within a 10cm ring situated around the circumscribing circle. Maximal outbound speed was computed similarly for the outbound path. Then the maximal outbound/inbound speeds' ratios for each excursion were computed. The dependency of maximal outbound/inbound speed's ratios (*r_n_*) on the ordinal number of the excursion (*n*) seemed to have a linear tendency. Therefore, we fitted a linear function of *n* to the *r_n_* in the form:
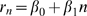
In this model β_0_ is the intercept and β_1_ is the slope.

## Supporting Information

Figure S1 Contour plots highlighting path curvature in the sessions of all saline-injected mice. Each horizontal line represents, from left to right, all the sessions of a mouse. The colors represent path curvature measured with a 20cm window. Locations without paths and locations with low path curvatures are colored in blue, medium curvature locations are colored in green, and high curvature locations are colored in red. Red high curvature patches along and near the wall reflect the prevalence of u-turns performed by the mice along the arena boundary. As shown, knots are established mainly at the location of the introduction into the arena (6 mice, 25 sessions, at about 12 o'clock position at a distance of 50 cm from wall), but also in other locations (near center toward 5 o'clock: mouse #1, session 1; #2, session 1, #3 session 2, #4 sessions 3,4,5,8; #8 sessions 2,3. near wall at 11 o'clock: mouse #2 session 4. Between center and 9 o'clock: mouse #7 session 10.) Some mice use only the place of entry for knot establishment (mouse #5) whereas others alternate between two (mice #1, #3, #8), or even 3 locations (mouse # 2). Every mouse established a knot during at least one session. The last session of the 5th mouse was not recorded because of a technical failure.
(8.29 MB TIF)

Figure S2 Timing and frequency of visits to knots in all saline-injected mice. Each plot shows the timing of visits to a knot across a single mouse- session. Visits to the knots are marked by vertical bars whose width is proportional to the visit's duration. Visits containing knot-scribbling are shown in red and all other visits in gray. As can be seen from the distribution of the red lines, knots are visited sporadically and frequently and visits include knot-scribbling throughout the session.
(2.31 MB TIF)

Figure S3 Contour plots representing the number of visits per location in all saline-injected mice. Each horizontal line represents, from left to right, all the sessions of a mouse. The colors represent the number of visits to locations (as defined by a 5×5cm grid). Locations with a low number of visits or no visits are colored in blue, locations with a medium number of visits are colored in green, and locations with a high number of visits are colored in red. Although, as with knots, a high number of visits was paid to the location of entry into the arena by a large number of mice, a high number of visits was also observed along the wall. While paying a high number of visits to knots, knot locations were not singled out by the number of visits paid to them (they were singled out only by knot-scribbling). The last session of the 5th mouse was not recorded because of a technical failure
(4.52 MB TIF)

Figure S4 Contour plots representing cumulative dwell time per location in all saline-injected mice. Each horizontal line represents, from left to right, all the sessions of a mouse. The colors represent dwell time. Low, medium and high dwell time locations are colored as in Figure S3. Although some mice spent a relatively long time at the location of entry (highlighted in green), places with the highest dwell time were located predominantly along the arena boundary, and thus knots, unlike home bases, were not characterized by the highest cumulative dwell time spent in them. The last session of the 5th mouse was not recorded because of a technical failure
(3.84 MB TIF)

Figure S5 Illustration of a knot established by a selected intact C57BL/6 mouse in a free setup allowing free passage between the mouse's home cage and a 2.5m diameter arena. A. Path traveled by the mouse within 1h during free exploration. B. Contour plot of path curvatures. Window size and colors are as in Figure S1. A knot is established at the 7 o'clock position. C. and D. show the path-scribbling performed within the knot during two separate visits paid to it (in black).
(2.44 MB TIF)
