# Supplementary figures and images for: Knots: Attractive Places with High Path Tortuosity in Mouse Open Field Exploration
Source: PLoS Comput Biol. 2010 Jan 15;6(1):e1000638. doi: 10.1371/journal.pcbi.1000638 (PMC2796396; doi:10.1371/journal.pcbi.1000638)

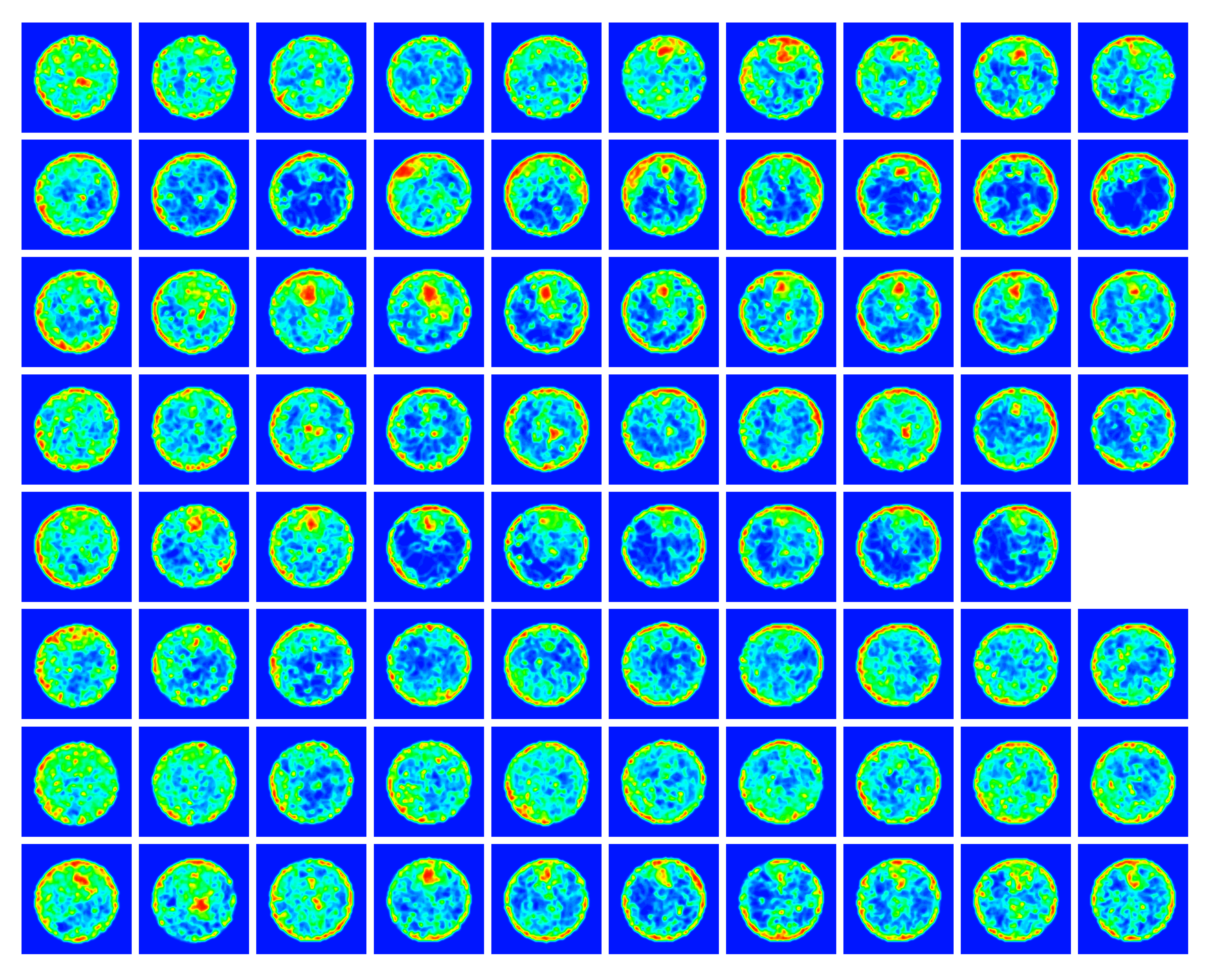

Supplement: Figure S1 — Contour plots highlighting path curvature in the sessions of all saline-injected mice. Each horizontal line represents, from left to right, all the sessions of a mouse. The colors represent path curvature measured with a 20cm window. Locations without paths and locations with low path curvatures are colored in blue, medium curvature locations are colored in green, and high curvature locations are colored in red. Red high curvature patches along and near the wall reflect the prevalence of u-turns performed by the mice along the arena boundary. As shown, knots are established mainly at the location of the introduction into the arena (6 mice, 25 sessions, at about 12 o'clock position at a distance of 50 cm from wall), but also in other locations (near center toward 5 o'clock: mouse #1, session 1; #2, session 1, #3 session 2, #4 sessions 3,4,5,8; #8 sessions 2,3. near wall at 11 o'clock: mouse #2 session 4. Between center and 9 o'clock: mouse #7 session 10.) Some mice use only the place of entry for knot establishment (mouse #5) whereas others alternate between two (mice #1, #3, #8), or even 3 locations (mouse # 2). Every mouse established a knot during at least one session. The last session of the 5th mouse was not recorded because of a technical failure. (8.29 MB TIF) [file pcbi.1000638.s001.tif]

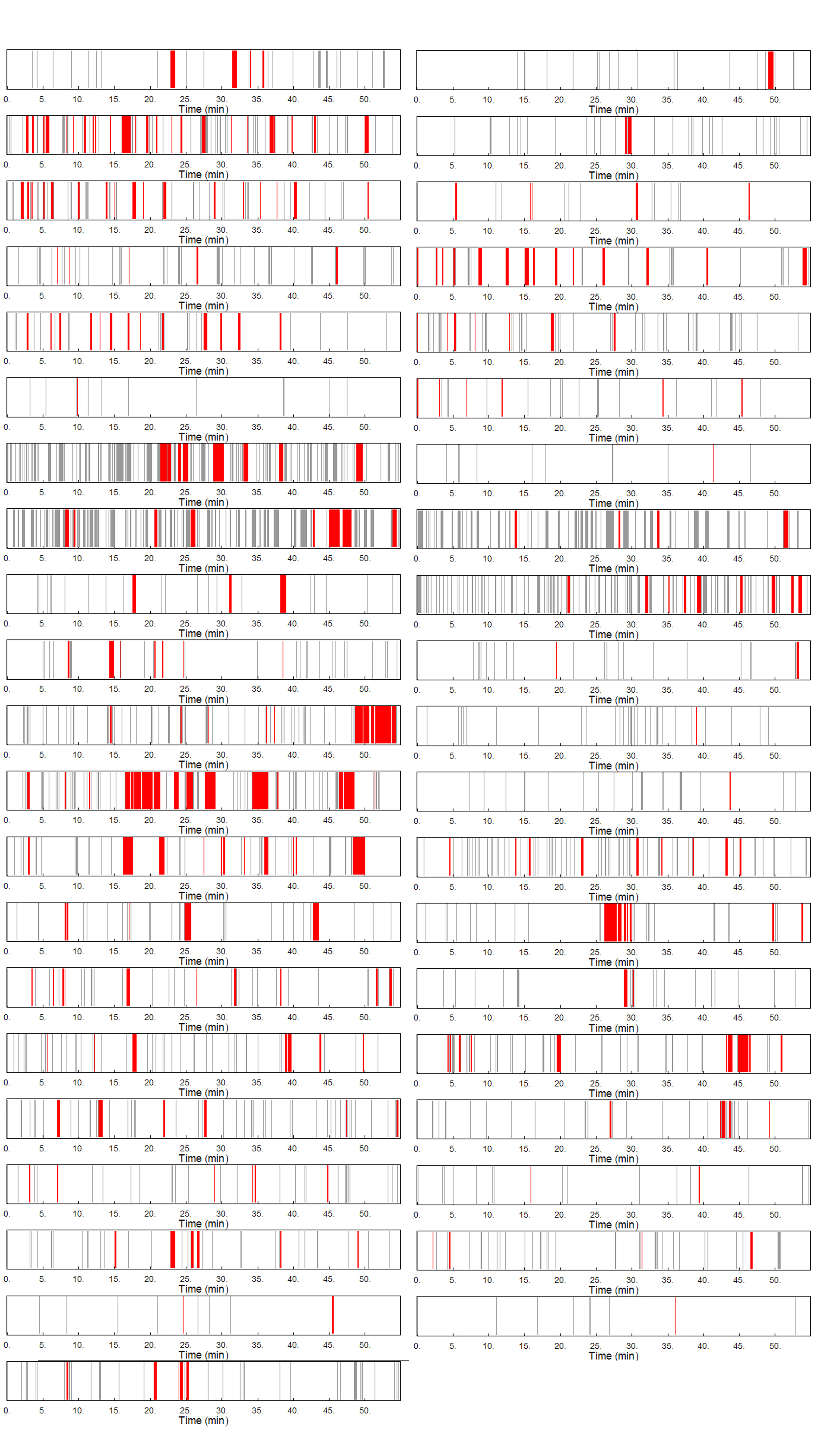

Supplement: Figure S2 — Timing and frequency of visits to knots in all saline-injected mice. Each plot shows the timing of visits to a knot across a single mouse- session. Visits to the knots are marked by vertical bars whose width is proportional to the visit's duration. Visits containing knot-scribbling are shown in red and all other visits in gray. As can be seen from the distribution of the red lines, knots are visited sporadically and frequently and visits include knot-scribbling throughout the session. (2.31 MB TIF) [file pcbi.1000638.s002.tif]

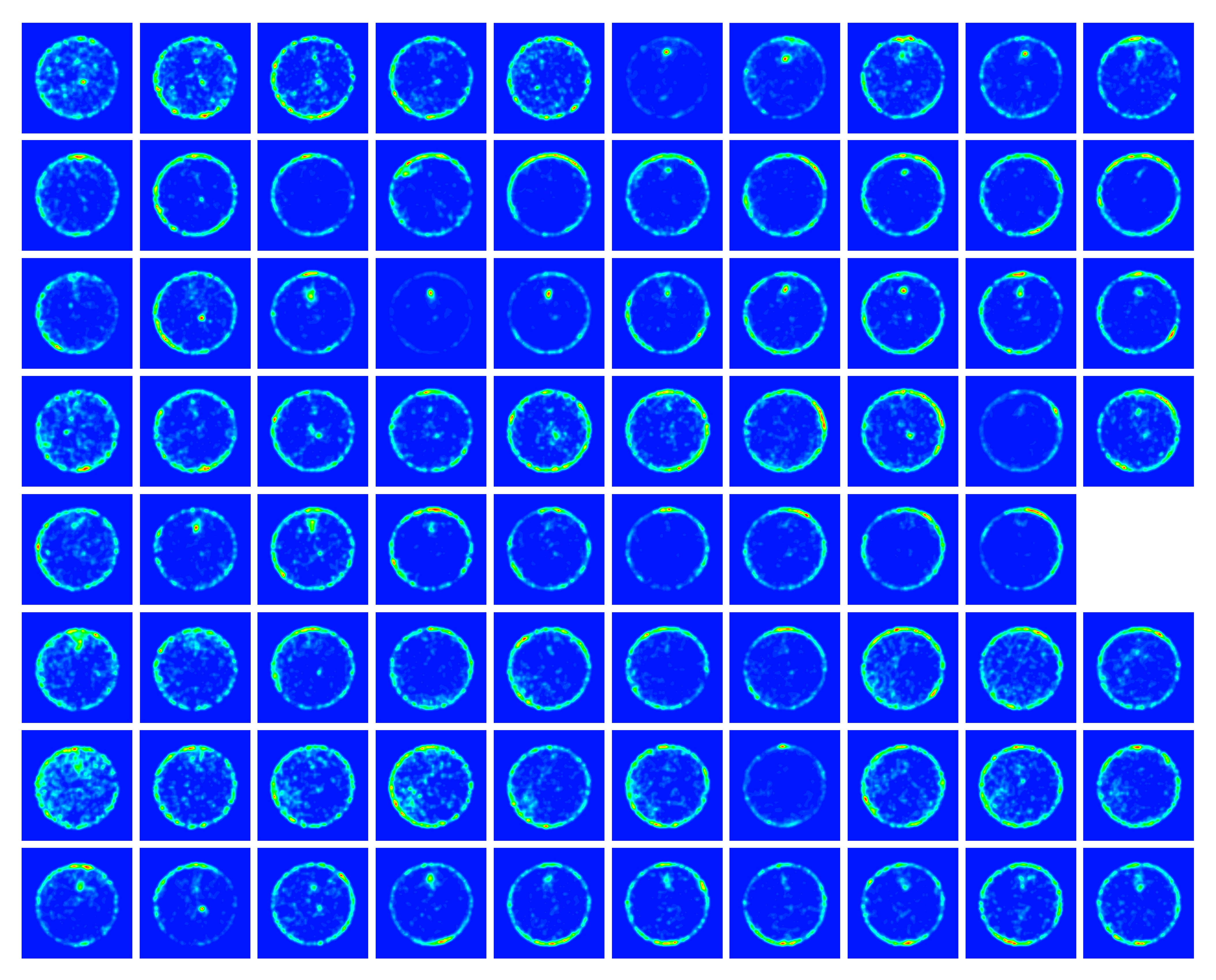

Supplement: Figure S3 — Contour plots representing the number of visits per location in all saline-injected mice. Each horizontal line represents, from left to right, all the sessions of a mouse. The colors represent the number of visits to locations (as defined by a 5×5cm grid). Locations with a low number of visits or no visits are colored in blue, locations with a medium number of visits are colored in green, and locations with a high number of visits are colored in red. Although, as with knots, a high number of visits was paid to the location of entry into the arena by a large number of mice, a high number of visits was also observed along the wall. While paying a high number of visits to knots, knot locations were not singled out by the number of visits paid to them (they were singled out only by knot-scribbling). The last session of the 5th mouse was not recorded because of a technical failure (4.52 MB TIF) [file pcbi.1000638.s003.tif]

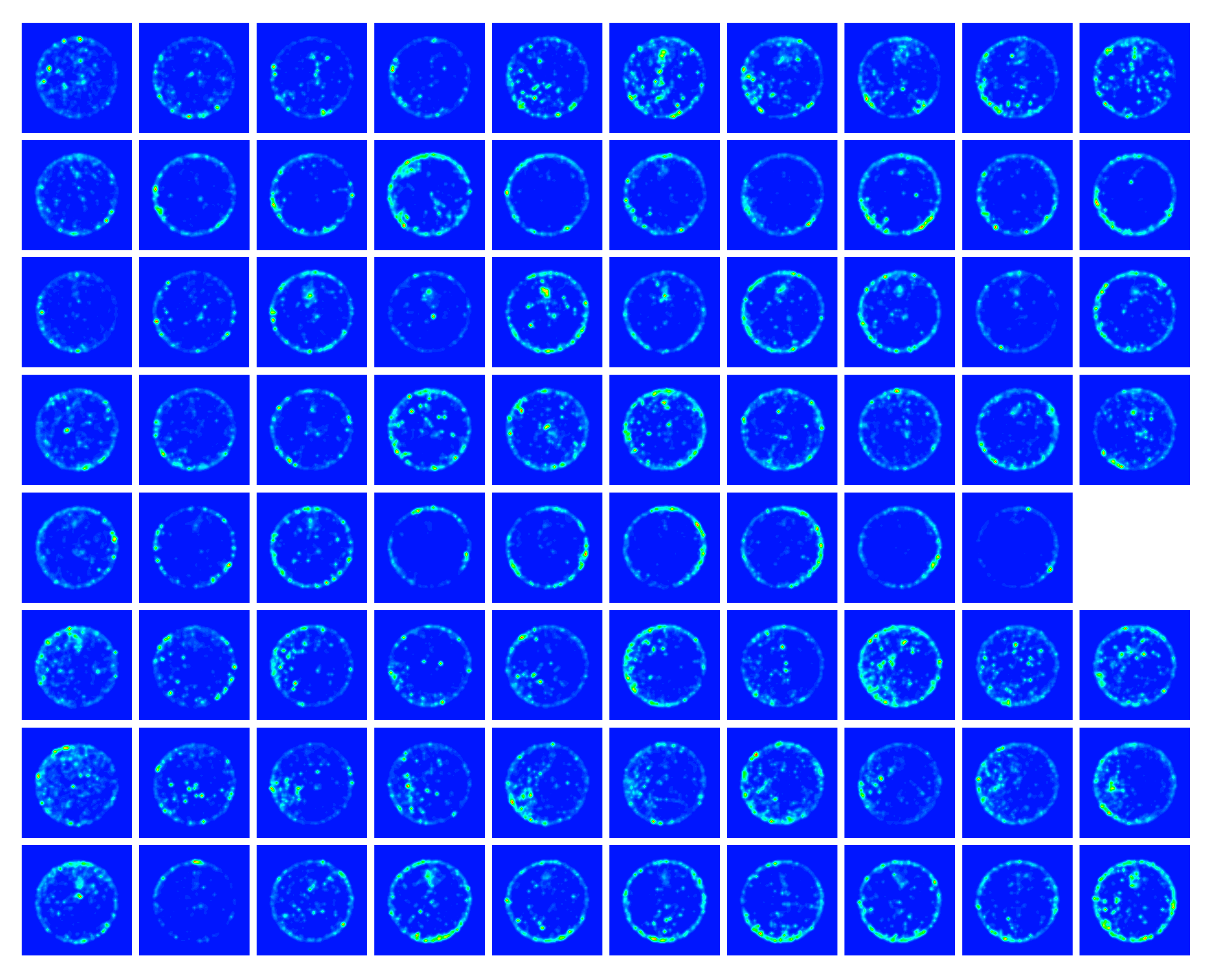

Supplement: Figure S4 — Contour plots representing cumulative dwell time per location in all saline-injected mice. Each horizontal line represents, from left to right, all the sessions of a mouse. The colors represent dwell time. Low, medium and high dwell time locations are colored as in Figure S3. Although some mice spent a relatively long time at the location of entry (highlighted in green), places with the highest dwell time were located predominantly along the arena boundary, and thus knots, unlike home bases, were not characterized by the highest cumulative dwell time spent in them. The last session of the 5th mouse was not recorded because of a technical failure (3.84 MB TIF) [file pcbi.1000638.s004.tif]

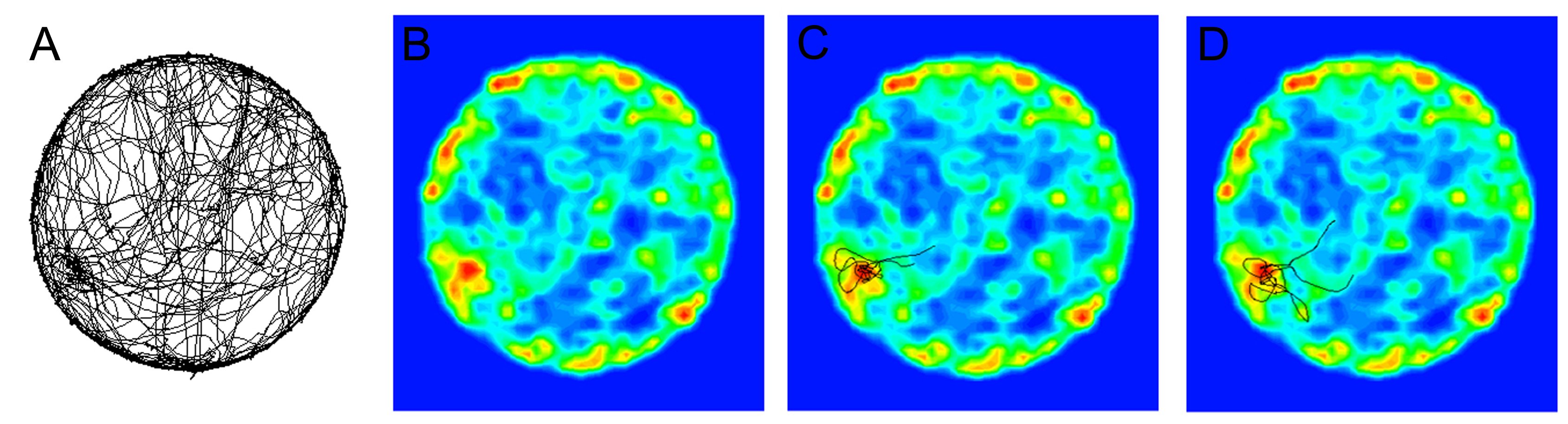

Supplement: Figure S5 — Illustration of a knot established by a selected intact C57BL/6 mouse in a free setup allowing free passage between the mouse's home cage and a 2.5m diameter arena. A. Path traveled by the mouse within 1h during free exploration. B. Contour plot of path curvatures. Window size and colors are as in Figure S1. A knot is established at the 7 o'clock position. C. and D. show the path-scribbling performed within the knot during two separate visits paid to it (in black). (2.44 MB TIF) [file pcbi.1000638.s005.tif]
